# Use of Response Surface Methodology in the Formulation and Optimization of Bisoprolol Fumarate Matrix Tablets for Sustained Drug Release

**DOI:** 10.5402/2012/730624

**Published:** 2012-12-10

**Authors:** Jadupati Malakar, Amit Kumar Nayak, Soumita Goswami

**Affiliations:** ^1^Department of Pharmaceutics, Bengal College of Pharmaceutical Sciences and Research, Durgapur, West Bengal 713212, India; ^2^Department of Pharmaceutics, Seemanta Institute of Pharmaceutical Sciences, Mayurbhanj, Odisha 757086, India

## Abstract

The aim of this investigation was to develop and optimize bisoprolol fumarate matrix tablets for sustained release application by response surface methodology based on 2^3^ factorial design. The effects of the amounts of calcium alginate, HPMC K4M, and Carbopol 943 in bisoprolol fumarate matrix tablets on the properties of bisoprolol fumarate sustained release matrix tablets like drug release and hardness were analyzed and optimized. The observed responses were coincided well with the predicted values by the experimental design. The optimized bisoprolol fumarate matrix tablets showed prolonged sustained release of bisoprolol fumarate over 6 hours. These matrix tablets followed the first-order model with anomalous (non-Fickian) diffusion mechanism.

## 1. Introduction

Matrix tablets offer a great potential as oral drug delivery system due to their simplicity, cost effectiveness, reduced risk of systemic toxicity, and minimal chance of dose dumping [1, 2]. They exclude complex production procedures such as coating and pelleatization during manufacturing, and drug release rate from the dosage form is controlled mainly by the type and proportion of polymer used in the preparations [3]. Various natural and synthetic hydrophilic polymers have been used in production of matrix tablets for soluble and insoluble drugs for years [1, 4–7]. Hydrophilic matrices are generally made of hydrogels, which have water absorbing capacity and swell to form a gel-like structure [8]. Swollen gel acts as a reservoir which slowly releases the drug dispersed already in the hydrogel matrices. Over the years, the use of polymer combinations as blends to prolong the drug release rate has become more popular, which may allow formulators to develop sustained release dosage forms that may show performance improvements over the individual polymer components [2]. Among various methods of the matrix tablet preparation, direct compression method has attracted much attention due to its technological simplicity and industrial acceptability as it involves simple blending of all ingredients used in formulations followed by compression. Moreover, it requires fewer unit operations, less machinery, reduced number of personnel and reduced processing time, increased product stability, and faster production rate [9].

**Scheme 1:**
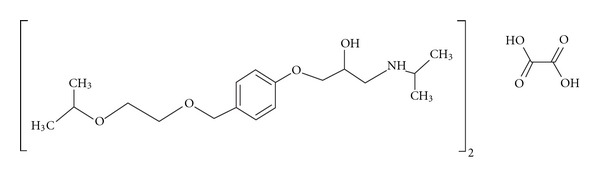
Chemical structure of bisoprolol fumarate.

Bisoprolol fumarate, chemically 1-[4-(2-isopropoxyethoxymethyl)phenoxy]-N-isopropyl-3-aminopropan-2-ol fumarate, is a cardioselective *β*-blocker (Scheme 1) without membrane stabilizing activity or intrinsic sympathomimetic activity [10, 11]. It is used for the treatment of hypertension and angina pectoris [11]. It is almost completely absorbed from the gastrointestinal tract and undergoes minimal first-pass metabolism resulting in an oral bioavailability of about 90%. However, multiple dosing of various conventional immediate release tablets is necessary to maintaine desired therapeutic level. Hence, sustained release formulations of bisoprolol fumarate could be beneficial to reduce the dosing frequency with improved patient compliance.

In the development of any pharmaceutical formulation like matrix tablet for sustained release ability, an important issue is to design a formulation with optimized quality in a short time period and minimum number of trials. The response surface methodology has been commonly used for designing and optimization of different pharmaceutical formulations, which requires minimum experimentation [12, 13]. Thus, it is less time-consuming and cost-effective than the conventional methods of formulating dosage forms. Based on the design of experiments, response surface methodology encompasses the generation of polynomial equations and of the response over the experimental domain to determine the optimum formulation(s) [12]. The objective of the current investigation was to develop bisoprolol fumarate sustained release matrix tablet containing a hydrophilic polymer-blend of calcium alginate, hydroxypropyl methylcellulose (HPMC K4M), and Carbopol 943 by direct compression method using response surface methodology. A computer-aided optimization technique using 2^3^ (three-factor and two-level) factorial design was employed to investigate the effect of the amounts of various hydrophilic polymers, namely, calcium alginate, HPMC K4M, and Carbopol 943 in polymer-blend used as three independent process variables (factors), on the properties of bisoprolol fumarate sustained release matrix tablets like drug release and hardness.

## 2. Materials and Methods

### 2.1. Materials

Bisoprolol fumarate, microcrystalline cellulose (PH 101), and lactose were obtained from B. S. Traders Pvt. Ltd., India. Calcium alginate, hydroxypropyl methylcellulose (HPMC K4M), and Carbopol 943 were purchased from Merck Specialties Pvt. Ltd., India. All other chemicals and reagents used were of analytical grade.

### 2.2. Preparation of Bisoprolol Fumarate Matrix Tablets

Bisoprolol fumarate matrix tablets were prepared by the direct compression method after proper mixing of suitable ratios of various hydrophilic polymers as release modifiers with others excipients. The drug, polymers, and other excipients were first passed through sieve #80. Then drug and all the materials were uniformly mixed and compressed on single punch tablet machine (Cadmach Machinery Co. Pvt. Ltd., India) using 6 mm round and flat punches (for batch size 100 tablets).

### 2.3. Experimental Design

2^3^ (three-factor and two-level) factorial design was employed for the optimization of bisoprolol fumarate matrix tablet. The amount of hydrophilic polymers in polymer-blend, namely, amount of calcium alginate (*A*), HPMC K4M (*B*), and Carbopol 943 (*C*) as the prime selected independent variables (factors), which were varied at two levels (low and high). Different trial formulations of bisoprolol fumarate matrix tablets were prepared according to the trial proposal of 2^3^ factorial design. The formulation chart for all proposed trial formulations is presented in Table 1. The cumulative drug release after 6 hours (*R*_6h_, %) and hardness (kg/cm^2^) was investigated as dependent variable (responses). Design-Expert 8.0.6.1 software (Stat-Ease Inc., USA) was used for generation and evaluation of the statistical experimental design. The matrix of the design including investigated factors and responses is shown in Table 2.

For optimization, the effects of independent variables upon the responses were modeled using the following first-order polynomial equations involving independent variables and their interactions for various measured responses, studied in this investigation. For optimization, effects of various independent variables upon measured responses were modeled using following mathematical model equation involving independent variables and their interactions for various measured responses generated by 2^3^ factorial design is as follows:

(1) Y=b0+b1A+b2B+b3C+b4AB+b5AC+b6BC,

where *Y* is the dependent variable, while *b*_o_ is the intercept; *b*_1_, *b*_2_, *b*_3_, *b*_4_, *b*_5_, *b*_6_, and *b*_7_ are regression coefficients; *A*, *B* and *C* are independable variables and *AB*,   *AC*, and *BC* are interactions between variables. One-way ANOVA was applied to estimate the significance of the model (*P* < 0.05) and individual response parameters.

### 2.4. Determination of Drug Content

20 tablets from each formulation batch were weighted and powdered. The powder equivalent to 20 mg of bisoprolol fumarate was taken and transferred to 100 mL of volumetric flask. Then, the volume was made up to 100 mL with 0.1 N HCl. Vigorous shaking was done to dissolve the powdered material in 0.1 N HCl. Samples were filtered using filter paper no. 40. After proper dilution, absorbance values were measured at the maximum wavelength (*λ*_max⁡_) using a UV-VIS spectrophotometer (Thermo Spectronic UV-1, USA) at 224 nm.

### 2.5. Weight Variation Determination

Tewenty tablets from each batch were sampled and accurately weighed using an electronic analytical balance. The weight variation (%) was calculated as

(2) Weight  variation  (%)=  Standard  deviationMean  weight×100.

### 2.6. Hardness Testing

Pfizer hardness tester was used to determine the hardness of prepared bisoprolol matrix tablets. The tablets were first kept in between two jaws after adjusting the tester to zero, a force was applied until the tablet breaks into fragments, and the reading was noted from the scale, which indicated the pressure required in kg.

### 2.7. *In Vitro* Drug Release Studies

*In Vitro *drug release studies were performed in USP dissolution apparatus, basket type (Campbell Electronics, India), at 50 rpm maintained at 37 ± 0.5°C. The various bisoprolol fumarate matrix tablets were placed into the basket. The dissolution mediums used were 900 mL of 0.1 N HCl (pH 1.2) for the first 2 hours and then 900 mL of phosphate buffer (pH 7.4) for next hours. The 5 mL of aliquots was withdrawn from the dissolution vessel at specific time intervals and replaced with equivalent volume of fresh medium. Collected dissolution samples were filtered using Whatman filter paper (no. 40) and then used for determination of bisoprolol fumarate spectrophotometrically using a UV-VIS spectrophotometer (Thermo Spectronic UV-1, USA) at 224 nm.

### 2.8. Kinetic Analysis of Release Data

To analyze the mechanism of drug release from these bisoprolol fumarate matrix tablets, the *in vitro *dissolution data were fitted to various mathematical models like zero-order, first-order, Higuchi, and Korsmeyer-Peppas models [14–17].

Zero-order model: *F* = *K*_0_ *t*, where *F* represents the fraction of drug released in time *t*, and *K*_0_ is the apparent release rate constant or zero-order release constant.

First-order model: ln⁡(1 − *F*) = −*K*_1st_ *t*, where *F* represents the fraction of drug released in time *t* and *K*_1_ is the first-order release constant.

Higuchi model: *F* = *K*_*H*_ *t* ^1/2^  , where *F* represents the fraction of drug released in time *t*, and *K*_*H*_ is the Higuchi dissolution constant.

Korsmeyer-Peppas Model: *F* = *K*_*P*_ *t* ^*n*^, where *F* represents the fraction of drug released in time *t*, *K*_*P*_ is the rate constant and *n* is the release exponent; this indicates the drug release mechanism.

Again, the Korsmeyer-Peppas model has been employed in the *in vitro *drug release behavior analysis of various pharmaceutical formulations to distinguish between various release mechanisms: Fickian release (diffusion-controlled release), non-Fickian release (anomalous transport), and case-II transport (relaxation-controlled release). When *n* ≤ 0.5, it is Fickian release. The *n* value between 0.5 and 1.0 is defined as non-Fickian release. When *n* ≥ 1.0, it is case-II transport and this involves polymer dissolution and polymeric chain enlargement or relaxation [17].

### 2.9. Statistical Analysis

Statistical optimization was performed using Design-Expert 8.0.6.1 software (Stat-Ease Inc., USA). All other data were analyzed with simple statistics.

## 3. Results and Discussions

### 3.1. Optimization of Bisoprolol Fumarate Matrix Tablets

Traditionally, pharmaceutical formulators develop various formulations by changing one variable at a time, and the method is time-consuming. However, many experiments, not succeed in their purpose because they are not properly thought out and designed, and even the best data analysis cannot compensate lack of planning. Therefore, it is essential to understand the influence of formulation variables on the quality of formulations with a minimal number of experimental trials and subsequent selection of formulation variables to develop an optimized formulation using established statistical tools for optimization [18]. A total of 8 trial formulations of bisoprolol fumarate matrix tablets were proposed by the 2^3^ factorial design for three independent variables: amounts of calcium alginate (*A*, mg), HPMC K4M (*B*, mg), and Carbopol 943 (*C*, mg), which were varied at two different levels (high and low). The effects of these independent variables on cumulative drug release after 6 hours (*R*_6h_), percentage and hardness, kg/cm^2^, were investigated as optimization response parameters in the current investigation. According to the 2^3^ factorial design, various trial formulations of bisoprolol fumarate matrix tablets were prepared by direct compression method using ingredients stated in Table 1. Overview of the experimental trial and observed responses is presented in Table 2. The results of the ANOVA indicated that these models were significant for all response parameters (Table 3). The Design-Expert 8.0.6.1 software provided suitable polynomial model equations involving individual main factors and interaction factors after fitting these data. The model equation relating *R*_6h_ as response became.

(3) R6h(%)=84.37−0.64A−0.87B−0.78C+6.5 ×10−3  AB+1.1×10−2AC+9.4×10−3BC,[R2=0.9999;  F-value=2714.99;  P<0.05].

The model equation relating hardness as response became:

(4) Hardness  (kg/cm2)    =2.84  +0.02A+7.87×10−3B   +0.05C+2.50×10−4AB−1.08   ×10−3AC−1.12×10−4BC  [R2=0.9996;  F-value=454.17;  P<0.05].

Model simplification was carried out by eliminating nonsignificant terms (*P* > 0.05) in model equations resulting from the multiple regression analysis [19], giving

(5) R6h(%)=84.37−0.64A−0.87B−0.78C  +9.4×10−3BC,Hardness  (kg/cm2)=2.84+0.02A+7.87×10−3B  +0.05C−1.08×10−3AC.

**Figure 1:**
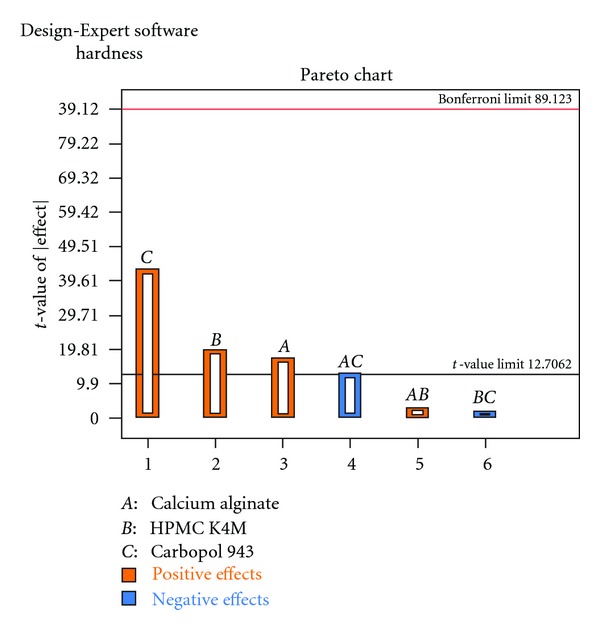
Pareto chart relating R_6h_ (%).

**Figure 2:**
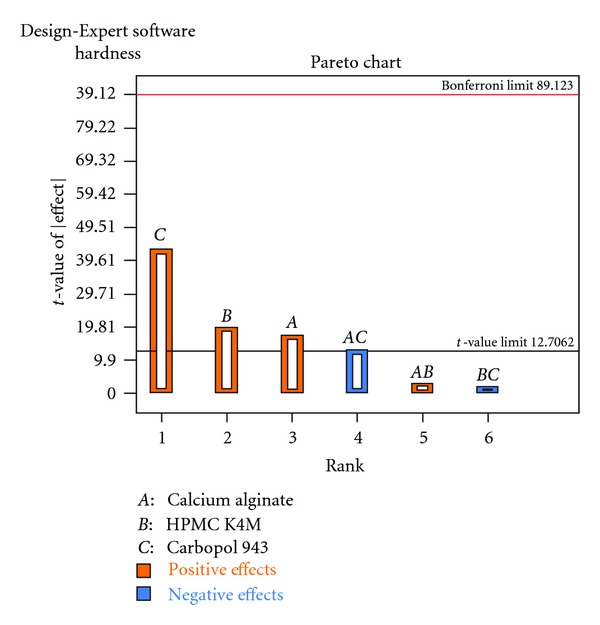
Pareto chart relating hardness (kg/cm^2^).

**Figure 3:**
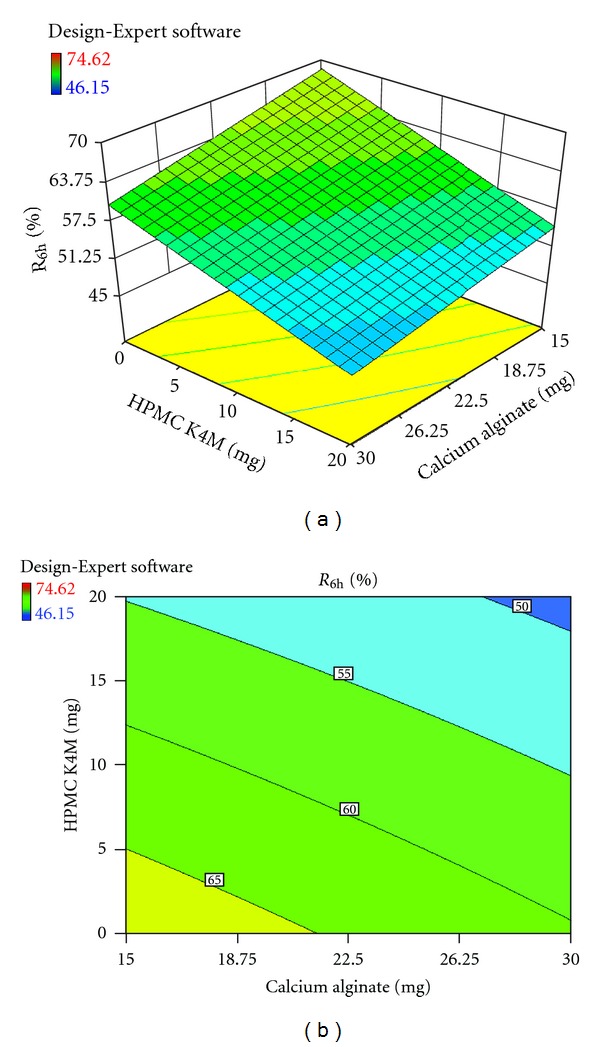
Effect of amounts of calcium alginate and HPMC K4M on R_6h_ (%) presented by response surface plot (a), and contour plot (b).

**Figure 4:**
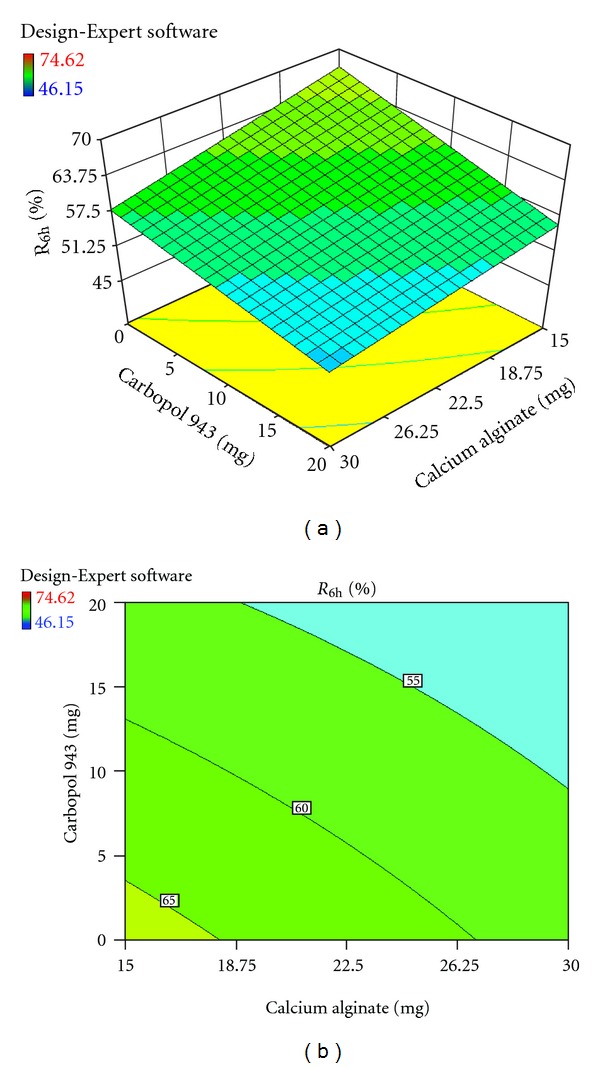
Effect of amounts of calcium alginate and Carbopol 943 on R_6h_ (%) presented by response surface plot (a), and contour plot (b).

**Figure 5:**
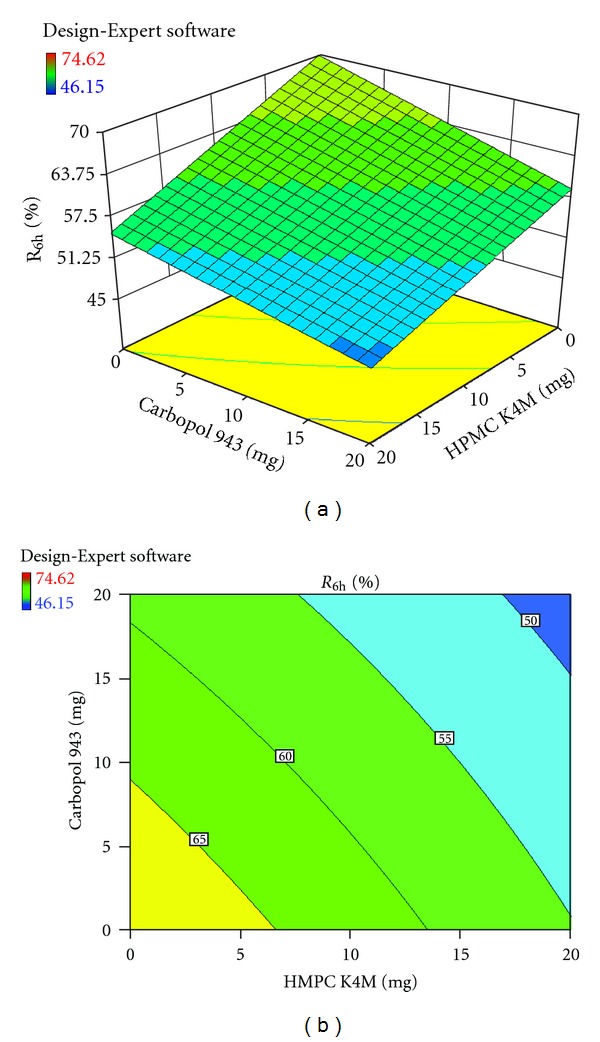
Effect of amounts of HPMC K4M and Carbopol 943 on *R*_6h_ (%) presented by response surface plot (a), and contour plot (b).

**Figure 6:**
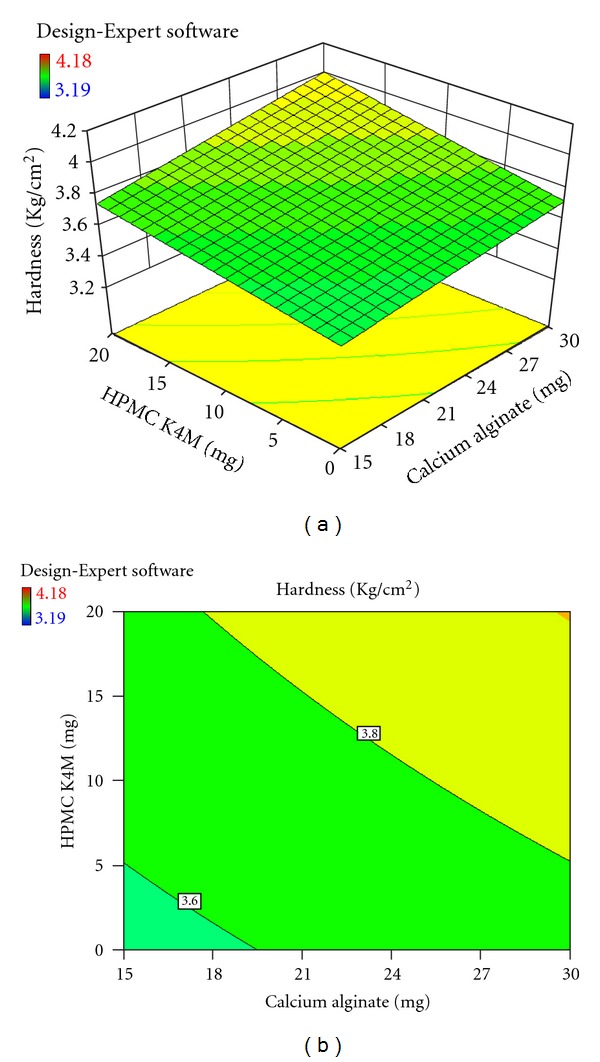
Effect of amounts of calcium alginate and HPMC K4M on hardness (kg/cm^2^) presented by response surface plot (a), and contour plot (b).

**Figure 7:**
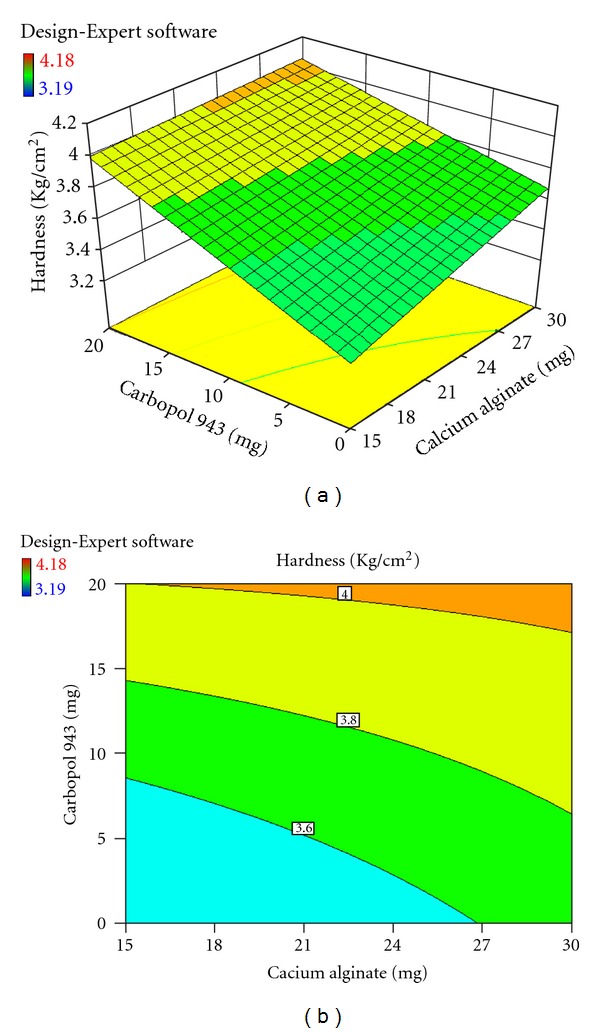
Effect of amounts of calcium alginate and Carbopol 943 on hardness (kg/cm^2^) presented by response surface plot (a), and contour plot (b).

**Figure 8:**
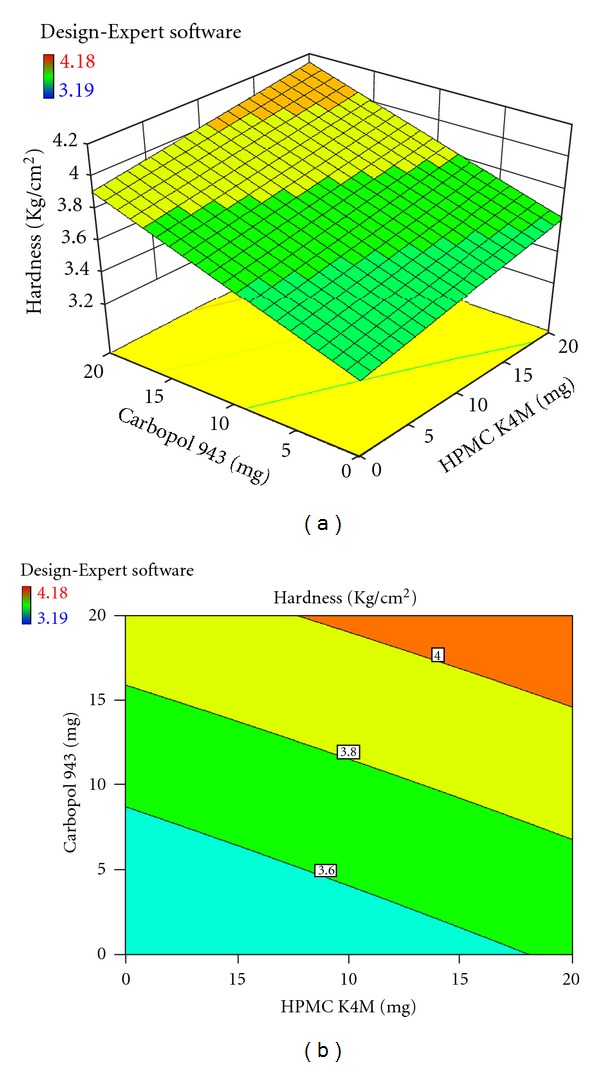
Effect of amounts of HPMC K4M and Carbopol 943 on hardness (kg/cm^2^) presented by response surface plot (a), and contour plot (b).

Each response coefficient was studied for its statistical significance by Pareto charts as shown in Figures 1 and 2. These charts depicted the statistical significance of each response coefficient. Coefficients with *t* values of effects above the Bonferroni line are designated as significant coefficients coefficients with *t* values of effects between Bonferroni line and *t* limit line are termed as coefficients likely to be significant; coefficients with *t* values of effects below the *t* limit line are statistically insignificant coefficients [20]. Therefore, these Pareto charts supported also the ANOVA results for the model simplification by eliminating nonsignificant terms (*P* > 0.05) in both the model equations. The influences of main effects (factors) on responses investigated (here, *R*_6h_, % and hardness, kg/cm^2^) were further elucidated by response surface methodology. Response surface methodology is a widely proficient approach in the development and optimization of drug delivery devices [12]. Three-dimensional response surface plots and their corresponding contour plots to estimate the effects of the independent variables (factors) on each response investigated were presented in Figures 3, 4, 5, 6, 7, and 8. The three-dimensional response surface plot is very useful in learning about the main and interaction effects of the independent variables (factors), whereas two-dimensional contour plot gives a visual representation of values of the response [18]. The three-dimensional response surface plots and corresponding contour plots relating *R*_6h_ and hardness indicate the deceased values of *R*_6h_ and increased values of hardness with the increment of all three independent variables (amounts of calcium alginate, HPMC K4M, and Carbopol 943 in bisoprolol fumarate matrix tablets).

A numerical optimization technique based on the desirability approaches was adopted to achieve new optimized formulation with desired responses. The desirable range of these responses was restricted to 40≤*R*_6h_ ≤ 60% and 4 ≤ hardness ≤ 5 kg/cm^2^, whereas the ranges of factors were restricted to 15 ≤ *A* ≤ 20 mg, 30 ≤ *B* ≤ 35 mg, and 30 ≤ *C* ≤ 35 mg. In order to evaluate the optimization capability of these models generated according to the results of 2^3^ factorial design, optimized bisoprolol matrix tablets were prepared by direct compression method using one of the selected optimal process variable settings proposed by the experimental design. The selected optimal process variable settings used for the formulation of optimized bisoprolol matrix tablets were *A* = 15.28 mg, *B* = 32.12 mg, and *C* = 30.31 mg. The numerical analysis was performed to acquire the optimal values of responses based on desirability criterion by the help of Design expert 8.0.6.1 software, which led to develop optimized bisoprolol fumarate matrix tablets (FO).  Table 4 depicts the results of predicted values obtained from the mathematical model and practically observed (actual value). The optimized bisoprolol fumarate matrix tablets (F-O) showed *R*_6h_ of 41.61 ± 1.97% and hardness of 4.65 ± 0.07 kg/cm^2^ within small error values (less than 5), indicating that mathematical models achieved from the 2^3^ factorial design were well fitted.

### 3.2. Drug Content and Weight Variation

All the bisoprolol fumarate matrix tablets contained bisoprolol fumarate within 97.99 ± 0.85 to 99.03 ± 0.59 (Table 5). Therefore, the drug content results ascertain the presence of bisoprolol fumarate in appropriate amount in all the matrix tablets. Weight variation does serve as a pointer to good manufacturing practices (GMPs) maintained by the manufacturers as well as amount of active pharmaceutical ingredients (APIs) contained in the formulation. All these bisoprolol fumarate matrix tablets met the USP specifications for weight uniformity [21]. The weight variation of these tablets varied from 1.88 ± 0.08 to 3.07 ± 0.28%, and none of the tablets deviated by up to 5% from their mean weight (Table 5). The uniform drug content and weight of the formulated bisoprolol fumarate matrix tablets indicate the uniform mixing of bisoprolol fumarate with other ingredients used.

### 3.3. Hardness

The hardness test for bisoprolol fumarate matrix tablets was done to assess the ability of tablets to withstand handling without fracturing or chipping. A force of about 4 kg/cm^2^ is the minimum requirement for a satisfactory hardness of tablets [22]. The results of the hardness testing showed that the hardness of all these matrix tablets was within the range between 3.19 ± 0.25 and 4.65 ± 0.07 kg/cm^2^ (Tables 2 and 4), indicating satisfactory hardness.

### 3.4. *In Vitro* Drug Release

**Figure 9:**
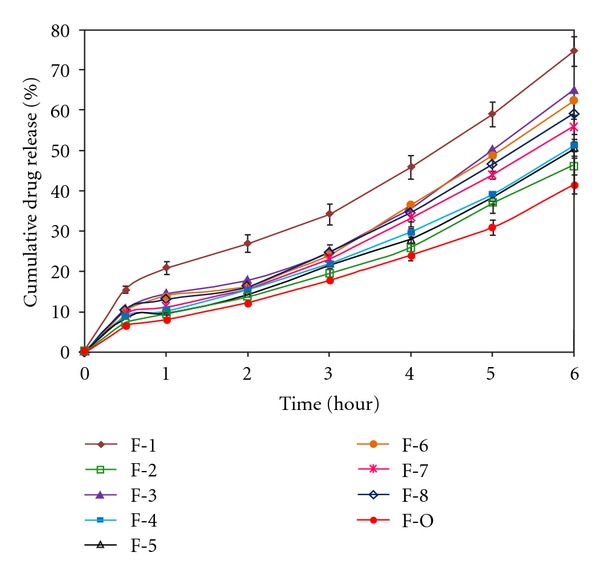
*In vitro *drug release from various bisoprolol fumarate matrix tablets (F-1 to F-O). Values are represented as (mean ± S.D., *n* = 3).

*In vitro *drug release from all bisoprolol fumarate matrix tablets was in the 0.1 N HCl (pH, 1.2) for the first 2 hours and then in phosphate buffer (pH, 7.4) for the next 4 hours. All these matrix tablets containing bisoprolol fumarate showed prolonged sustained drug release over 6 hours (Figure 9). The cumulative drug release from these matrix tablets after 6 hours of dissolution (*R*_6h_, %) was within the range, 41.61 ± 1.97 to 74.62 ± 2.36%. From the response surface analysis, it was found that the *R*_6h_ values decreased with the increment of all three independent variables (amount of calcium alginate, HPMC K4M, and Carbopol 943). The higher viscosity due to increasing amount of various hydrophilic polymers used, calcium alginate, HPMC K4M, and Carbopol 943 may promote the formation of highly viscous gels upon contact with aqueous fluids of the dissolution medium, which would retard the drug release rate from these bisoprolol fumarate matrix tablets.

The *in vitro *drug release data from various bisoprolol fumarate matrix tablets were evaluated kinetically using various mathematical models such as zero-order, first-order, Higuchi, and Korsmeyer-Peppas models. The results of the curve fitting into these above-mentioned mathematical models are given in Table 6. When respective correlation coefficients of drug release from bisoprolol fumarate matrix tablets were compared, it was found to follow the first-order model (*R* ^2^ = 0.9906  to  0.9953) over a period of 6 hours. The value of release exponent (*n*) determined from *in vitro *bisoprolol fumarate release data of various matrix tablets ranged from 0.56 to 0.74, indicating anomalous (non-Fickian) diffusion mechanism of drug release. The anomalous diffusion mechanism of drug release demonstrates both diffusion-controlled and swelling-controlled drug release from bisoprolol fumarate matrix tablets.

## 4. Conclusion

Bisoprolol fumarate matrix tablets for sustained release application were successfully developed by response surface methodology based on 2^3^ factorial design. The amounts of calcium alginate, HPMC K4M, and Carbopol 943 in bisoprolol fumarate matrix tablets on the properties of bisoprolol fumarate sustained release matrix tablet like drug release and hardness were analyzed and optimized. The three-dimensional response surface plots and corresponding contour plots relating *R*_6h_ and hardness indicate the deceased values of *R*_6h_ and increased values of hardness with the increment of amount of calcium alginate, HPMC K4M, and Carbopol 943. The optimized bisoprolol fumarate matrix tablets (F-O) showed *R*_6h_ of 41.61 ± 1.97% and hardness of 4.65 ± 0.07 kg/cm^2^. These developed optimized matrix tablets showed prolonged sustained release of bisoprolol fumarate over 12 hours, which might be beneficial over the conventional tablet to reduce the dosing frequency with improved patient compliance.

## Figures and Tables

**Table 1:** The formulation chart for all proposed trial formulations of bisoprolol fumarate matrix tablets.

Formulation codes	Drug (mg)	Calcium alginate (mg) (*A*)	HPMC K4M (mg) (*B*)	Carbopol 943 (mg) (*C*)	Lactose (mg)	MCC (mg)^a^	Mg-stearate (mg)
F-1	20	15 (−1)	0 (−1)	0 (−1)	70	20	10
F-2	20	30 (+1)	20 (+1)	20 (+1)	70	20	10
F-3	20	30 (+1)	0 (−1)	0 (−1)	70	20	10
F-4	20	30 (+1)	20 (+1)	0 (−1)	70	20	10
F-5	20	15 (−1)	20 (+1)	20 (+1)	70	20	10
F-6	20	15 (−1)	0 (−1)	20 (+1)	70	20	10
F-7	20	30 (+1)	0 (−1)	20 (+1)	70	20	10
F-8	20	15 (−1)	20 (+1)	0 (−1)	70	20	10

^a^MCC: Microcrystalline cellulose; *X*_1_, *X*_2_, and *X*_3_ represent the main effects (factors); (+1): higher value and (−1): lower value.

**Table 2:** 2^3^ factorial designs and their observed response values with drug contents in bisoprolol fumarate matrix tablets.

Formulation codes	Calcium alginate (mg) *A*	HPMC K4M (mg) *B*	Carbopol 943 (mg) *C*	Responses	
				*R*_ 6h _(%)^a,b^	Hardness (kg/cm^2^)^b^
F-1	15 (−1)	0 (−1)	0 (−1)	74.62 ± 2.36	3.19 ± 0.25
F-2	30 (+1)	20 (+1)	20 (+1)	46.15 ± 2.12	4.18 ± 0.15
F-3	30 (+1)	0 (−1)	0 (−1)	65.07 ± 3.54	3.52 ± 0.20
F-4	30 (+1)	20 (+1)	0 (−1)	51.32 ± 3.25	3.84 ± 0.15
F-5	15 (−1)	20 (+1)	20 (+1)	50.39 ± 2.16	4.10 ± 0.10
F-6	15 (−1)	0 (−1)	20 (+1)	62.33 ± 1.92	3.90 ± 0.30
F-7	30 (+1)	0 (−1)	20 (+1)	55.85 ± 2.22	3.93 ± 0.10
F-8	15 (−1)	20 (+1)	0 (−1)	59.17 ± 1.82	3.41 ± 0.25

^a^ *R*_ 6h _(%): cumulative drug release after 6 hours; ^b^mean ± S.D., *n* = 6; *A*, *B*, and *C* represent the main effects (factors); (+1): higher value and (−1): lower value.

**Table 3:** Summary of ANOVA for response parameters.

Source	Sum of square	d.f.^a^	Mean square	*F* value	*P* value prob > *F*
For *R*_6h_ (%)^b^					
Model	593.77	6	98.96	2714.99	0.0147 (S)
*A*	98.84	1	98.84	2711.71	0.0122 (S)
*B*	323.09	1	323.09	8863.87	0.0068 (S)
*C*	157.18	1	157.18	4312.11	0.0097 (S)
*AB*	1.94	1	1.94	53.24	0.0867 (NS)
*AC*	5.58	1	5.58	153.08	0.0514 (NS)
*BC*	7.14	1	7.14	196.00	0.0454 (S)

For hardness (kg/cm^2^)					
Model	0.85	6	0.14	454.17	0.0359 (S)
*A*	0.10	1	0.10	302.76	0.0365 (S)
*B*	0.12	1	0.12	392.04	0.0321 (S)
*C*	0.58	1	0.58	1849.00	0.0148 (S)
*AB*	2.81 × 10^−3^	1	2.81 × 10^−3^	9.00	0.2048 (NS)
*AC*	0.05	1	0.05	169.00	0.0489 (S)
*BC*	1.01 × 10^−3^	1	1.01 × 10^−3^	3.24	0.3228 (NS)

^a^d.f. indicates degree of freedom; ^b^ *R*_6h _(%): cumulative drug release after 6 hours; *A*, *B*, and *C* represent the main effects (factors)—the amount of calcium alginate, HPMC K4M, and Carbopol 943 in mg, respectively; *AB*, *AC*, and *BC* are their interaction effects; S and NS indicate significance and nonsignificance, respectively.

**Table 4:** Results of experiments to assure optimization capability.

Code	Calcium alginate (mg) *A*	HPMC K4M (mg) *B*	Carbopol 943 (mg) *C*	Responses	
				*R*_ 6h_ (%)^a^	Hardness (kg/cm^2^)
F-O	15.28	32.12	30.31	Actual values^b^	
				41.61 ± 1.97	4.65 ± 0.07
				Predicted values	
				40.00	4.54

% Error^c^				4.03	2.42

^a^ *R*_ 6h_ (%): cumulative drug release after 6 hours; ^b^actual values = mean ± S.D., *n* = 3; ^c^% error = (actual value − predicted value)/predicted value × 100; *A*, *B*, and *C* represent the main effects (factors).

**Table 5:** Drug content and weight variation of bisoprolol fumarate matrix tablets.

Formulation codes	Drug content (%)^a^	Weight variation (%)^b^
F-1	99.03 ± 0.59	2.12 ± 0.18
F-2	98.42 ± 0.72	2.25 ± 0.24
F-3	98.14 ± 0.65	3.04 ± 0.28
F-4	97.99 ± 0.85	3.07 ± 0.19
F-5	99.11 ± 1.22	2.31 ± 0.15
F-6	98.62 ± 0.52	1.90 ± 0.12
F-7	98.92 ± 0.78	2.02 ± 0.14
F-8	98.66 ± 0.55	1.92 ± 0.13
F-O	98.93 ± 0.72	1.88 ± 0.08

^a^Mean ± S.D., *n* = 20; ^b^coefficient of weight variation (%) = standard deviation/mean weight × 100.

**Table 6:** Results of curve fitting of the *in vitro* bisoprolol release data from different bisoprolol fumarate matrix tablets.

Formulation code	Correlation coefficient (*R* ^2^)				Release exponent (*n*)
	Zero-order	First-order	Higuchi	Korsmeyer-Peppas
F-1	0.9744	0.9908	0.8279	0.9410	0.60
F-2	0.9698	0.9929	0.7067	0.9415	0.73
F-3	0.9467	0.9951	0.6842	0.8979	0.69
F-4	0.9710	0.9948	0.7286	0.9317	0.70
F-5	0.9632	0.9941	0.7060	0.9083	0.70
F-6	0.9566	0.9906	0.6924	0.9047	0.71
F-7	0.9673	0.9953	0.7058	0.9092	0.71
F-8	0.9650	0.9944	0.7212	0.9099	0.69
F-O	0.9733	0.9910	0.7111	0.9460	0.74
